# Response to a case report: Idiopathic hypereosinophilic syndrome in remission with benralizumab treatment after relapse with mepolizumab

**DOI:** 10.1002/rcr2.707

**Published:** 2021-01-04

**Authors:** Gema Requena, Florence Roufosse, Lee D. Baylis, Jonathan Steinfeld

**Affiliations:** ^1^ Epidemiology, Value Evidence and Outcomes Global R&D, GSK London UK; ^2^ Department of Internal Medicine, Hôpital Erasme Université Libre de Bruxelles Brussels Belgium; ^3^ Respiratory Medical Franchise GSK Research Triangle Park NC USA; ^4^ Respiratory Research & Development GSK Collegeville PA USA

## Correspondence

*To the Editor*:

We read with interest the case report by Fujii et al. regarding an 83‐year‐old male patient with relapsing idiopathic‐hypereosinophilic syndrome (I‐HES) after treatment with mepolizumab [1]. There are some discrepancies in this case report that should be noted and require further clarification. First, the dose of mepolizumab was not specified for this patient throughout the paper. This is important as the only available dose in Japan is 100 mg subcutaneous (SC), which is licensed for asthma, and the dose used in the HES phase 3 clinical trial was 300 mg SC every four weeks (NCT02836496). This is also the dose the US Food and Drug Administration (FDA) recently approved for treatment of HES [2]. If the patient was dosed with less than 300 mg SC every four weeks, then he was undertreated and the treatment effect of mepolizumab was potentially underestimated. In addition, the patient's full medical history was not given, which could have important implications on the cause and course of his eosinophilia.

Furthermore, the authors have used data on the clinical effect of mepolizumab in mild atopic asthma to make assumptions around its efficacy in the treatment of HES. They state that mepolizumab “selectively inhibits eosinophilic inflammation while reducing eosinophil content in the sputum and blood” but that “this holds true only in 50% of the airway tissues and bone marrows and has no significant effect on bronchial mucosal staining of the eosinophil major basic protein.” The paper referenced for this assertion is a study [3] in patients aged 18–55 years with mild atopic asthma, not patients with HES as this case report suggests. Biopsy data from patients with HES are not available.

As shown in figure 2 of the referenced Fujii et al.'s paper, the reduction in blood eosinophilic count in this patient appears to show an equivalent reduction in eosinophil counts for both mepolizumab and benralizumab. Despite this, the authors go on to state that benralizumab is more effective at depleting eosinophils in tissues by citing one study, again in patients with asthma, not HES. They then state that “benralizumab produces a median decrease in the content of peripheral blood eosinophils from a baseline of 95.8% in the airway mucosa, 89.9% in sputum, and 100% in the bone marrow,” which appears to confuse peripheral blood eosinophils with mucosal, sputum, and bone marrow eosinophils. Also, no lung biopsy or bronchoalveolar lavage was performed in the cited study nor in this patient. We are not aware of any direct head‐to‐head comparisons in similar patient populations of mepolizumab and benralizumab with respect to eosinophil tissue depletion.

Whilst we welcome the sharing of clinical experience with biologic agent therapy in this difficult‐to‐treat clinical scenario, it is premature to formulate any conclusion on whether mepolizumab or benralizumab is more effective in HES based on a single case report, especially when key information is omitted. With the recent US approval of mepolizumab in HES, more observations and data could be gathered in real‐world observational studies to address this interesting issue.

## Disclosure Statements

FR reports consultancy fees from AstraZeneca, GSK, and Knopp Biosciences. JS, LDB, and GR are all employees of GSK and own stocks/shares.
